# Erythromycin-resistant lactic acid bacteria in the healthy gut of vegans, ovo-lacto vegetarians and omnivores

**DOI:** 10.1371/journal.pone.0220549

**Published:** 2019-08-02

**Authors:** Vesna Milanović, Andrea Osimani, Federica Cardinali, Alice Litta-Mulondo, Carla Vignaroli, Barbara Citterio, Gianmarco Mangiaterra, Lucia Aquilanti, Cristiana Garofalo, Francesca Biavasco, Luca Cocolin, Ilario Ferrocino, Raffaella Di Cagno, Silvia Turroni, Camilla Lazzi, Nicoletta Pellegrini, Francesca Clementi

**Affiliations:** 1 Dipartimento di Scienze Agrarie, Alimentari ed Ambientali, Università Politecnica delle Marche, Ancona, Italy; 2 Dipartimento Scienze della Vita e dell’Ambiente, Università Politecnica delle Marche, Ancona, Italy; 3 Department of Biomolecular Sciences, Biotechnology Section, University of Urbino ‘Carlo Bo’, Urbino, Italy; 4 Department of Agricultural, Forest and Food Science (DISAFA), University of Turin, Grugliasco, Italy; 5 Faculty of Science and Technology, Libera Università di Bolzano, Bolzano, Italy; 6 Department of Pharmacy and Biotechnology, Alma Mater Studiorum, University of Bologna, Bologna, Italy; 7 Department of Food Science, University of Parma, Parma, Italy; University of Messina, ITALY

## Abstract

Diet can affect the diversity and composition of gut microbiota. Usage of antibiotics in food production and in human or veterinary medicine has resulted in the emergence of commensal antibiotic resistant bacteria in the human gut. The incidence of erythromycin-resistant lactic acid bacteria (LAB) in the feces of healthy vegans, ovo-lacto vegetarians and omnivores was analyzed. Overall, 155 LAB were isolated and characterized for their phenotypic and genotypic resistance to erythromycin. The isolates belonged to 11 different species within the *Enterococcus* and *Streptococcus* genera. *Enterococcus faecium* was the dominant species in isolates from all the dietary categories. Only 97 out of 155 isolates were resistant to erythromycin after Minimum Inhibitory Concentration (MIC) determination; among them, 19 isolates (7 from vegans, 4 from ovo-lacto vegetarians and 8 from omnivores) carried the *erm*(B) gene. The copresence of *erm*(B) and *erm*(A) genes was only observed in *Enterococcus avium* from omnivores. Moreover, the transferability of erythromycin resistance genes using multidrug-resistant (MDR) cultures selected from the three groups was assessed, and four out of six isolates were able to transfer the *erm*(B) gene. Overall, isolates obtained from the omnivore samples showed resistance to a greater number of antibiotics and carried more tested antibiotic resistance genes compared to the isolates from ovo-lacto vegetarians and vegans. In conclusion, our results show that diet does not significantly affect the occurrence of erythromycin-resistant bacteria and that commensal strains may act as a reservoir of antibiotic resistance (AR) genes and as a source of antibiotic resistance spreading.

## Introduction

The intestinal tract is the largest reservoir of microbes in the human body, containing up to 10^14^ microbial cells of over 500 different species [1, 2]. Microorganisms associated with the human intestinal tract are referred to as the “gut microbiota,” which plays an important role in both the health and disease of the host through its impact on metabolism, nutrition, physiology, immunology and pathogenesis [3]. The composition of the gut microbiota is influenced by many factors, such as age, sex, host genetics and environment. Among environmental factors, diet exerts the largest impact on the human gut microbiota. Three main types of diet are currently recognized throughout the world, namely, omnivore, ovo-lacto vegetarian and vegan, each of which is a source of nutrients for microorganisms and is itself a reservoir of microbes that impose a huge influence on the abundance and diversity of gut microbiota [4]. The effect of long term and short term diet on the composition of the human gut microbiota has been extensively studied over the last decade [4, 5, 6, 7, 8, 9]. Furthermore, as reported by Hu et al. [10], the human gut contains the largest pool of antibiotic resistance (AR) genes (gut “resistome”) compared with other natural environments, such as soil or aquatic environments. This is of great concern since the emergence of AR is considered a large global health problem [11]. It has been reported that antimicrobial usage causes selection for and enhancement of antibiotic resistant bacteria in the human gut [12]. Among environmental factors, food and beverages represent the main reservoirs of antibiotic resistant bacteria and their AR genes for the human gut, which is characterized by the coexistence of numerous commensal, saprophytic, and even pathogenic bacterial species [13, 14, 15, 16]. This is not surprising considering the extensive administration of antibiotics in livestock, agriculture and aquaculture for several purposes, including therapy, prophylaxis and growth promotion [15]. Furthermore, fruits and vegetables may be exposed to antibiotic resistant bacteria from different sources, such as irrigation water, wastewater, contaminated manure or animals (wild and domestic) during growing in the field, harvesting or post-harvesting, transport and storage [17, 18].

Many previous studies have focused on AR genes in clinically relevant bacterial species [19, 20, 21]. However, further attention is currently being paid to the AR genes in foodborne and gut commensal bacteria, such as lactic acid bacteria (LAB), which may horizontally transfer their AR genes to either close or distantly related strains, including human pathogens in the gut [22, 23, 24]. The high cell density present in the human gut might favor the horizontal transfer of AR genes through some of the three major mechanisms, such as conjugation, natural transformation and transduction. Among these, conjugation is recognized as the most common mechanism of horizontal gene transfer in natural environments, including the human intestinal tract [25].

Nonetheless diet exerts a huge impact on the human gut microbiota, little is known on the effect of diet, especially long term diet, on its AR gene reservoir (“resistome”). The results reported in several previous studies [26, 27, 28, 29] were inconsistent, thus encouraging further investigation into this important topic. In our previous work [23], the small effect of long-term vegan, ovo-lacto vegetarian or omnivore diet on the prevalence of 12 AR genes conferring resistance to erythromycin, tetracyclines, vancomycin and β-lactams was seen after direct PCR screening of 144 fecal samples. Overall, the genes conferring resistance to erythromycin and tetracyclines were detected at the highest frequency. Since erythromycin-resistant genes are reported to be among the most widespread AR determinants in foodborne LAB [30], the aim of the present study was to evaluate the impact of different dietary habits on the distribution of erythromycin-resistant LAB strains in the feces of omnivores, vegetarians and vegans that showed a high frequency of erythromycin-resistant genes in a previous work [23]. All the isolates were subjected to genotypic and phenotypic determination of antibiotic resistance. Moreover, conjugation experiments were performed to evaluate the possible transmission of erythromycin-resistant genes from isolated multidrug-resistant (MDR) LAB strains to different recipients.

## Materials and methods

### Recruitment of participants following an omnivore, vegan or ovo-lacto vegetarian diet

144 healthy participants who were habitually following a long term (at least 1 year before recruiting) omnivore, ovo-lacto vegetarian or vegan diet were recruited from 4 different locations in Italy (Bari, Bologna, Parma and Turin) between February and July 2013. The recruited volunteers were asked to record the exact quantities (g or mL) of all the foods and beverages consumed daily during three consecutive weeks. An ovo-lacto vegetarian diet was assumed when the participants stated they did not consume any meat, fish and seafood, whereas a vegan diet was assumed for those participants who stated that they did not consume any foods from animal origin. The details about recruitment procedure as well as the exclusion criteria have been previously reported [5, 6, 23].

### Isolation of erythromycin-resistant lactic acid bacteria

Fecal samples from 144 healthy individuals with different diets (48 omnivores, 48 ovo-lacto vegetarians and 48 vegans) previously screened by PCR for the occurrence of 12 antibiotic resistance genes [23] were further analyzed to isolate erythromycin-resistant LABs. After defrosting, 1 g of each fecal sample was transferred in 9 mL of peptone water (1 g L^-1^ of peptone) and homogenized by vortexing. Aliquots of the homogenates were inoculated onto MRS agar (VWR Chemicals, Radnor, Pennsylvania, US) supplemented with erythromycin (4 mg L^-1^) (Sigma-Aldrich, Saint Louis, MO, USA). After 48–72 h of incubation at 30°C in anaerobic jars, presumptive erythromycin-resistant colonies were randomly selected and amplified on MRS agar plates supplemented with the same concentration of erythromycin used in the selection.

### Species identification

DNA from the isolates was extracted following the procedure described by Osimani et al. [31], resuspended at a concentration of 25 ng μL^-1^ and used in PCR assays.

The 16S rRNA gene was amplified by PCR with universal primers 27F (5’-AGAGTTTGATCCTGGCTCAG-3’) and 1495R (5’- CTACGGCTACCTTGTTACGA-3’) [32]. 2 μL (50 ng) of the extracted DNA was amplified in a total reaction volume of 50 μL containing 1U of Taq DNA polymerase (SybEnzyme, Novosibirsk, Russia), 1X reaction buffer, 0.2 mM deoxynucleoside triphosphates and 0.2 μM of each primer. The PCR was carried out using the amplification conditions previously described by Aquilanti et al. [33].

The obtained 16S rRNA amplicons (1468 bp) were digested separately with 2.5 U of the restriction enzymes *Alu*I, *Fok*I and *Hae*III (New England Biolabs, Ipswich, MA, USA) in 12.5 μL reaction volume. The reactions were performed at 37°C for 15 h for the preliminary identification of the isolates by Amplified Ribosomal DNA Restriction Analysis (ARDRA). 12.5 μL of each digested product were analyzed trough electrophoresis on a 2% (w/v) agarose gel (Conda pronadisa, Spain) in 0.5X TBE (45 mM Tris-borate, 1 mM EDTA) containing ethidium bromide (0.5 μg mL^-1^) at 75 V for 2 h. A 100-bp ladder (HyperLadder, Bioline, London, UK) was used as molecular weight standard. Gels were visualized under UV light and photographed with the Complete Photo XT101 system (Explera, Jesi, Italy). The comparison of banding profiles among different isolates was performed manually. Isolates showing identical restriction patterns were grouped together. To perform the species identification, 16S rRNA amplicons of one or more isolates from each ARDRA group were sent to Genewiz (Takeley, UK) for purification and sequencing. The obtained sequences in FASTA format were compared with the sequences deposited in the GenBank DNA database (http://www.ncbi.nlm.nih.gov/) using the Basic local Alignment Search Tool (BLAST) [34].

### Antimicrobial susceptibility testing and antibiotic resistance gene detection

Antibiotic susceptibility was assessed by determination of the minimum inhibitory concentration (MIC) according to the Clinical and Laboratory Standards Institute [35] guidelines, and the results were interpreted according to the CLSI breakpoints [36]. *Enterococcus faecalis* ATCC 29212 was used as a quality control strain. The resistance genes *erm*(A), *erm*(B) and *erm*(C) were assessed by PCR using previously described primers and conditions [23]. Isolates that were positive for one or more *erm* genes were selected and further analyzed for antimicrobial susceptibility to additional antibiotics by MIC determination as described above. The following antibiotics were tested: ampicillin (AMP), gentamicin (CN), streptomycin (STR), levofloxacin (LEV), quinupristin/dalfopristin (Q/D), linezolid (LZD), vancomycin (VAN), daptomycin (DAP) and tetracycline (TET), all of which were purchased from Sigma-Aldrich except for Q/D (Pfizer, Rome, Italy). Tetracycline [*tet*(M), *tet*(L), *tet*(O), *tet*(K)], gentamicin [*aac(6′)-Ie-aph(2″)-Ia*] and streptomycin [*ant(6’)-Ia*] resistance genes were detected by PCR as previously described [37, 38].

### Conjugation experiments

Conjugation experiments were performed by filter mating as previously described [39] using strains carrying multiple resistance genes isolated from the feces of omnivore (*E*. *faecium* 37BA and *E*. *faecalis* 37TO), ovo-lacto vegetarian (*E*. *faecalis* 13BA and *E*. *hirae* 01BA) and vegan (*E*. *faecalis* 27TO and *E*. *avium* 35PA) individuals as donors. *E*. *faecalis* JH2-2, *E*. *faecium* 64/3, both resistant to fusidic acid and rifampicin, were used as recipients.

Selection of transconjugants was performed on Brain Heart Infusion agar (BHIA) plates supplemented with erythromycin (20 μg mL^-1^), fusidic acid (10 μg mL^-1^) and rifampicin (10 μg mL^-1^). The conjugation frequency was expressed as the number (CFU mL^-1^) of transconjugants per recipient cell (CFU mL^-1^). Each experiment was performed in triplicate, and transconjugant frequencies were reported as the mean of three experiments. The relationship between the transconjugants and the relevant recipient was verified by species-specific PCR assays targeting genes coding for the D-ala-d-ala ligase (*ddl E*. *faecium* and *ddl E*. *faecalis*) or by the comparison of pulsed field gel electrophoresis (PFGE) profiles; both assays were performed as reported by Vignaroli et al. [37]. The stability of erythromycin resistance in the transconjugants was assessed by serial daily passages on antibiotic-free BHIA for a month. Every week, some colonies were tested for erythromycin susceptibility by MIC determination and by PCR detection of *erm*(B) gene.

### Statistical analysis

To compare the distribution of isolates within the different enterococcal species in the three categories of individuals (omnivores, ovo-lacto vegetarians and vegans) and to determine if there was a significant difference between the expected and the observed frequencies in each category, Chi-squared (χ^2^) test was performed. Also the prevalence of resistant strains and *erm*(B) genes among the three dietary groups were analyzed by the χ^2^ test. The significance was set at p < 0.05.

## Results and discussion

### Identification of isolates

In total, 155 isolates grown on MRS agar supplemented with erythromycin (4 mg L^-1^) were recovered from the fecal samples of the volunteers following an omnivore (50 isolates), ovo-lacto vegetarian (51 isolate) or vegan (54 isolates) diet. The collected isolates were grouped into 11 different profiles by ARDRA and identified to the species level on the basis of the 16S rRNA sequencing of the amplicon of one or more isolates of each group (S1 Table). All tested isolates belonged to *Enterococcus* or *Streptococcus* spp. This is not surprising since the LAB belonging to *Enterococcus* and *Streptococcus* genus are commensal members of the normal human gut microbiota [40, 41, 42]. Moreover, *Enterococcus* are widely distributed in a variety of foods of both animal and plant origin, likely due to their high resistance to adverse environmental conditions in food processing facilities and during storage. These bacteria can be used as a food contamination indicator or they can even play a beneficial role during the ripening of fermented foods by enhancing the flavor [43]. Two species, namely, *Enterococcus faecalis* and *Enterococcus faecium*, are the most prevalent species of this genus found in foods as well as in human feces [41]. The high incidence of resistance to different classes of antibiotics in enterococci isolated from different foods (including meat, dairy products, and vegetables) or used as probiotic strains is of great concern since, once ingested, they can survive gastric passage and act as a reservoir of AR genes in the human gut [12, 15].

**Fig 1:**
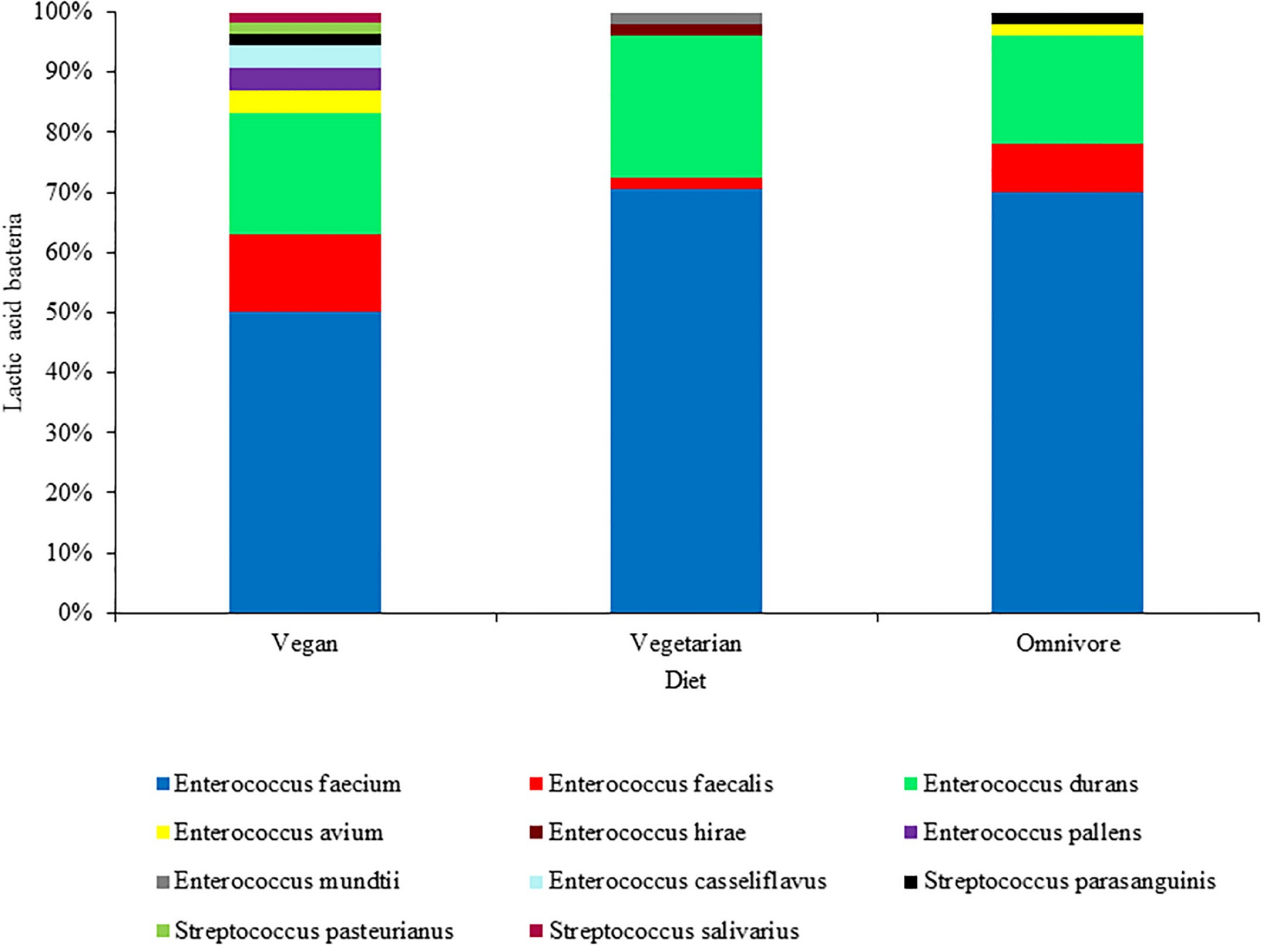
Distribution of lactic acid bacteria isolated from the fecal samples of healthy vegans, ovo-lacto vegetarians and omnivores grown on MRS agar plates supplemented with erythromycin (4 mg L^-1^).

The 155 LAB isolates obtained from MRS agar plates supplemented with erythromycin (4 mg L^-1^) were distributed among three dietary groups, as reported in Fig 1. *E*. *faecium* was the dominant species in all three categories, representing 70.6%, 70.0% and 50.0% of the total number of isolates from the feces of ovo-lacto vegetarians, omnivores and vegans, respectively. *Enterococcus durans* represented 23.5% of the isolates from ovo-lacto vegetarians, 20.4% of the isolates from vegans and 18% of the isolates from omnivores, while *E*. *faecalis* represented 13.0% of vegan, 8.0% of omnivore and 2.0% of vegetarian isolates. Other enterococcal species less frequently isolated from humans were recovered from the different fecal samples. *Enterococcus hirae* and *Enterococcus mundtii* were isolated exclusively from ovo-lacto vegetarian samples, each representing 2.0% of the isolates. *Enterococcus avium* was isolated from omnivore (2.0%) and vegan samples (3.7%), while *Enterococcus pallens* and *Enterococcus casseliflavus* were isolated only from vegan samples, both covering 3.7% of total isolates. The higher prevalence of *E*. *durans*, *E*. *mundtii*, *E*. *hirae* and *E*. *casseliflavus* species in fecal samples from vegans and ovo-lacto vegetarians might be related to their abundance in foods of plant origin, as reported in previous studies [44, 45, 46, 47].

The species from *Streptococcus* genera were isolated from vegan and omnivore samples. In detail, *Streptococcus pasteurianus* and *Streptococcus salivarius* were isolated exclusively from vegan samples in low proportions (1.9%), while the species *Streptococcus parasanguinis* was isolated from vegan and omnivore samples, covering 1.9% and 2.0% of total species, respectively. These species are part of the viridans group of streptococci, which can be both commensal and pathogenic in humans, colonizing the gastrointestinal and urinary tracts as well as the oral cavity mucosa [48]. However, statistical analysis showed no significant differences in the species distribution among the different dietary groups. This is in accordance with the previous study performed by Ferrocino et al. [6], in which the microbiota of the same fecal samples used in our work were studied by a combination of culture-dependent (viable counts on different culture media) and -independent methods (ribosomal RNA Denaturing Gradient Gel Electrophoresis), showing a high similarity of the fecal microbiota for the three investigated diets. Regarding the species diversity, vegan fecal samples were characterized by the highest richness in LAB species (9) compared to ovo-lacto vegetarian and omnivore samples with 5 different LAB species each (Fig 1). The prevalence of *E*. *faecium* compared to the minor enterococcal species (*E*. *avium*, *E*. *hirae*, *E*. *pallens*, *E*. *mundtii*, *E*. *casseliflavus*) was significant (*p* = 0.04) in the vegan group only.

### Erythromycin resistance and *erm* gene detection

**Fig 2:**
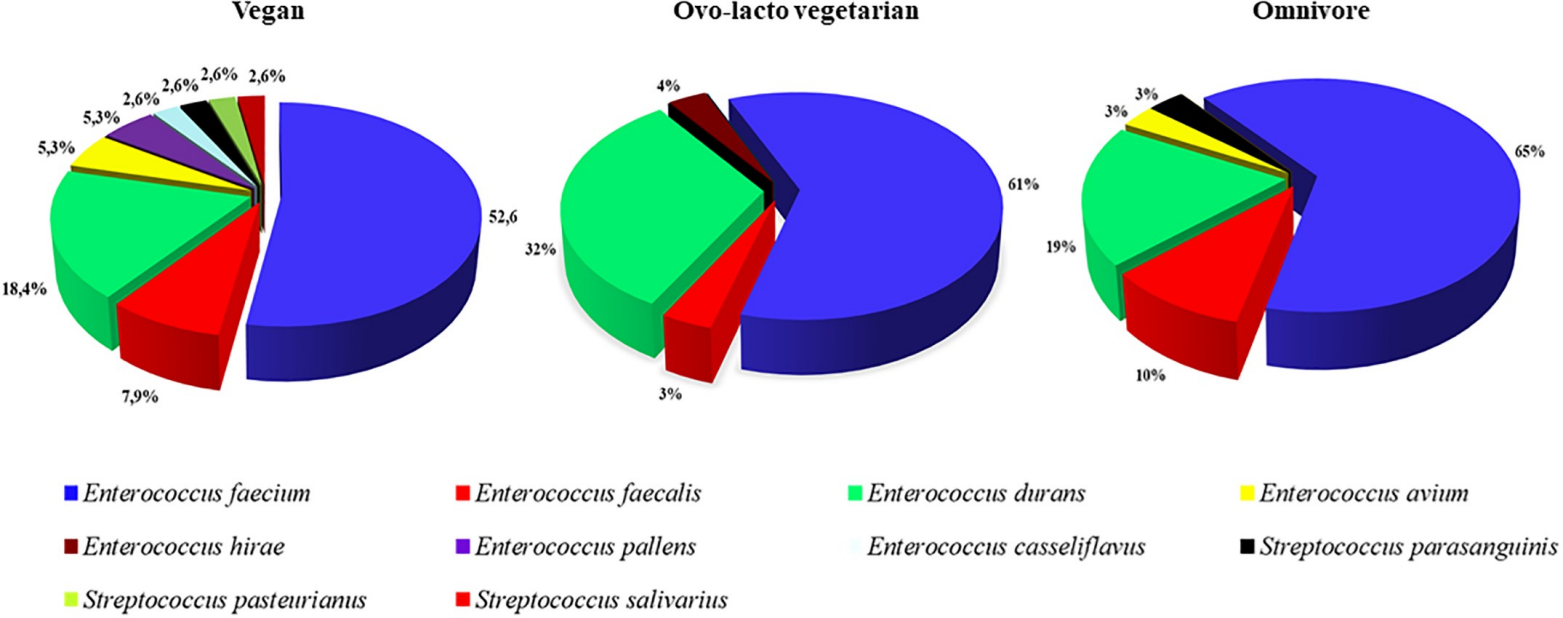
Distribution of phenotypically erythromycin-resistant lactic acid bacteria isolated from the fecal samples of healthy vegans, ovo-lacto vegetarians and omnivores.

All the isolates were subjected to erythromycin MIC determination. The results are summarized in Table 1, while the detailed results are shown in S1 Table. According to the CLSI (2017) breakpoints for resistance to erythromycin, *Enterococcus* and *Streptococcus* isolates were considered to be phenotypically resistant when the detected MIC values were ≥ 8 mg L^-1^ and ≥ 1 mg L^-1^, respectively. Not all the isolates grown on erythromycin containing MRS plates were phenotypically resistant to this antibiotic after MIC determination. Resistance to erythromycin was confirmed in 93 *Enterococcus* (35 from vegans, 28 from ovo-lacto vegetarians and 30 from omnivores) and 4 *Streptococcus* (3 from vegans and 1 from omnivores) strains (Table 1, S1 Table). The distribution of erythromycin-resistant species among three dietary groups is reported in Fig 2. The prevalence of isolates within the different enterococcal species in the three categories was not statistically significant (p>0.05).

**Table 1:** Prevalence and distribution of the MICs and erythromycin resistant genes among the lactic acid bacteria isolated from the fecal samples of vegans, ovo-lacto vegetarians and omnivores.

Species	Diet	Total number of tested strains	MIC values (μg mL^-1^) ^a^^,^ ^b^												Breakpoint (μg mL^-1^) *	Total number of phenotypically erythromycin-resistant strains	Erythromycin resistance genes		
			≤0.25	0.5	1	2	4	8	16	32	64	128	256	>256			*erm*(A)	*erm*(B)	*erm*(C)
*Enterococcus faecium*	Vegan	27	4	-	-	1	2	2	12	6	-	-	-	-	≥8	20 (74.1%)	-	-	-
	Vegetarian	36	1	5	2	6	5	1	8	6	1	-	-	1		17 (47.2%)	-	1	-
	Omnivore	35	-	1	2	6	6	8	5	2	1	-	-	4		20 (57%)	-	4	-
*Enterococcus faecalis*	Vegan	7	-	1	-	3	-	-	-	-	-	-	-	3	≥8	3 (42.9%)	-	3	-
	Vegetarian	1	-	-	-	-	-	-	-	-	-	-	-	1		1 (100%)	-	1	-
	Omnivore	4	-	1	-	-	-	-	-	-	-	-	-	3		3 (75%)	-	3	-
*Enterococcus durans*	Vegan	11	2	1	-	-	1	1	5	1	-	-	-	-	≥8	7 (63.6%)	-	0	-
	Vegetarian	12	-	-	-	1	2	2	4	1	1	-	-	1		9 (75%)	-	1	-
	Omnivore	9	-	-	2		1	4	-	1	1	-	-	-		6 (66.7%)	-	0	-
*Enterococcus hirae*	Vegan	-	-	-	-	-	-	-	-	-	-	-	-	-	≥8	-	-	0	-
	Vegetarian	1	-	-	-	-	-	-	-	1	-	-	-	-		1 (100%)	-	1	-
	Omnivore	-	-	-	-	-	-	-	-	-	-	-	-	-		-	-	0	-
*Enterococcus avium*	Vegan	2	-	-	-	-	-	-	-	-	-	1	-	1	≥8	2 (100%)	-	2	-
	Vegetarian	-	-	-	-	-	-	-	-	-	-	-	-	-		-	-	0	-
	Omnivore	1	-	-	-	-	-	-	-	-	-	-	-	1		1 (100%)	1	1	-
*Enterococcus pallens*	Vegan	2	-	-	-	-	-	-	1	-	1	-	-	-	≥8	2 (100%)	-	-	-
	Vegetarian	-	-	-	-	-	-	-	-	-	-	-	-	-		-	-	-	-
	Omnivore	-	-	-	-	-	-	-	-	-	-	-	-	-		-	-	-	-
*Enterococcus mundtii*	Vegan	-	-	-	-	-	-	-	-	-	-	-	-	-	≥8	-	-	-	-
	Vegetarian	1	-	1	-	-	-	-	-	-	-	-	-	-		-	-	-	-
	Omnivore	-	-	-	-	-	-	-	-	-	-	-	-	-		-	-	-	-
*Enterococcus casseliflavus*	Vegan	2	1	-	-	-	-	-	1	-	-	-	-	-	≥8	1 (50%)	-	-	-
	Vegetarian	-	-	-	-	-	-	-	-	-	-	-	-	-		-	-	-	-
	Omnivore	-	-	-	-	-	-	-	-	-	-	-	-	-		-	-	-	-
*Streptococcus pasteurianus*	Vegan	1	-	-	-	-	-	-	-	-	-	-	-	1	≥1	1 (100%)	-	1	-
	Vegetarian	-	-	-	-	-	-	-	-	-	-	-	-	-		-	-	-	-
	Omnivore	-	-	-	-	-	-	-	-	-	-	-	-	-		-	-	-	-
*Streptococcus parasanguinis*	Vegan	1	-	-	-	-	-	1	-	-	-	-	-	-	≥1	1 (100%)	-	-	-
	Vegetarian	-	-	-	-	-	-	-	-	-	-	-	-	-		-	-	-	-
	Omnivore	1	-	-	-	-	1	-	-	-	-	-	-	-		1 (100%)	-	-	-
*Streptococcus salivarius*	Vegan	1	-	-	-	-	-	-	-	-	-	-	-	1	≥1	1 (100%)	-	1	-
	Vegetarian	-	-	-	-	-	-	-	-	-	-	-	-	-		-	-	-	-
	Omnivore	-	-	-	-	-	-	-	-	-	-	-	-	-		-	-	-	-

* Clinical Laboratory Standard Institute breakpoints (CLSI, 2017);

^a^ overall MIC50: vegans (16 μg mL^-1^), ovo lacto vegetarians (8 μg mL^-1^), omnivores (8 μg mL^-1^);

^b^ overall MIC90: vegans (128 μg mL^-1^), ovo lacto vegetarians (32 μg mL^-1^), omnivores (>256 μg mL^-1^);

MIC, Minimum Inhibitory Concentration

Eight highly resistant strains with MICs >256 mg L^-1^ were isolated from omnivore samples, represented by 4 *E*. *faecium*, 3 *E*. *faecalis* and 1 *E*. *avium* isolate, while six and three highly resistant isolates were recovered from vegans (3 *E*. *faecalis*, 1 *E*. *avium*, 1 *S*. *pasteurianus* and 1 *S*. *salivarius*) and ovo-lacto vegetarians (1 *E*. *faecium*, 1 *E*. *faecalis* and 1 *E*. *durans*) samples (Table 1, S1 Table). Overall, the MIC_50_ of the total isolates from the three dietary groups were similar (16, 8 and 8 mg L^-1^ for vegan, vegetarian, and omnivore isolates, respectively), whereas the MIC_90_ was slightly higher for omnivore isolates (>256 mg L^-1^) than for vegan and vegetarian isolates (128 and 32 mg L^-1^, respectively).

The prevalence of highly erythromycin-resistant strains in the fecal samples collected from omnivores could be a consequence of excessive use of antibiotics in the breeding of farm animals. Although the use of antibiotics to promote the growth of farm animals has been forbidden in Europe since 2006, their use for therapeutic and prophylactic purposes is still permitted [49].

The highest, although not significant frequency of erythromycin-resistant strains among vegans (70%) respect ovo-lacto vegetarians (55%) or omnivores (62%), was unexpected (S2 Table). Food of vegetable origin could thus represent an underestimated source of AR in humans. Notably, the large use of antibiotics in food animals and aquaculture has resulted in the increase of resistant bacteria in the environment, soil and water bodies, and consequently in the possible contamination of crops and vegetables [15, 49]. Regarding the *Streptococcus* genus, *S*. *pasteurianus* and *S*. *salivarius* strains isolated from vegan samples showed a high level of resistance (>256 mg L^-1^) to erythromycin. The high rates of macrolide resistance among viridans group streptococci have been previously reported by Brenciani et al. [50]. This group of microorganisms uses the target site modification and macrolide efflux systems as the main mechanisms of MLS_B_ resistance [51].

The 97 erythromycin-resistant strains (38 from vegans, 28 from ovo-lacto vegetarians and 31 from omnivores) were screened for the occurrence of three erythromycin resistance genes, namely, *erm*(A), *erm*(B) and *erm*(C). As shown in Table 1 and S1 Table, only 19 isolates (7 from vegans, 4 from ovo-lacto vegetarians and 8 from omnivores) carried an *erm* gene; in particular, 18 carried *erm*(B), and only one (33TO from omnivores) carried both *erm*(B) and *erm*(A) genes. No significant differences were observed in the *erm*(B) distribution among the different dietary groups. The prevalence of the *erm*(B) gene among other *erm* genes was also confirmed in this study since this is the most frequently detected gene in LAB isolated from human stool [52, 23] as well as from different food samples [53]. The higher prevalence of *erm*(B) could be related to its frequent localization on mobile conjugative transposons [54] (i.e. Tn916-Tn1545 family) or mobilizable plasmids, as described for the *E*. *faecium* 37BA from omnivores [55]. The detection of a low number of genes conferring resistance to erythromycin among phenotypically resistant isolates could also be because we only analyzed the most common genes found in LAB, suggesting the possibility that these bacteria contain other genes involved in erythromycin resistance. The occurrence of the genes was very well correlated with the MIC results since almost all the isolates carrying *erm* genes were characterized by high-level resistance (MICs >256 mg L^-1^). Only one *E*. *hirae* strain (01BA from ovo-lacto vegetarians) with an MIC of 32 mg L^-1^ and one *E*. *avium* strain (24BA from vegans) with an MIC of 128 mg L^-1^ were positive for the genes. Highly erythromycin-resistant strains (MIC >256 μg mL^-1^) slightly prevailed among the omnivore samples. Furthermore, the copresence of *erm*(B) and *erm*(A) was detected in an omnivore strain.

Our findings are in accordance with the results obtained by Portillo et al. [56], who screened different *Enterococcus* strains for the presence of *erm*(A), *erm*(B) and *erm*(C) genes. *erm*(B) gene was detected in 98% of highly erythromycin-resistant *Enterococcus* isolates with MIC > 128 mg L^-1^, whereas enterococcal isolates for which erythromycin MICs were <32 mg L^-1^ were negative for *erm* methylase genes.

### Characterization of the selected erythromycin-resistant strains

The 19 *erm* positive strains were further analyzed for their resistance to additional antibiotics by MIC determination and PCR detection of the relevant resistance genes. Most of them were resistant to TET (16 out of 19) and STR (10 out of 19) in accordance to previous studies [55, 57, 58, 59], reporting that co-resistance to ERY, TET and STR in *E*. *faecium* and *E*. *faecalis* is frequently related to multi-resistant plasmids. Overall, isolates obtained from the omnivore samples were resistant to a greater number of antibiotics (up to 4) with respect to those from vegan samples. Moreover, omnivore samples carried more tested antibiotic resistance genes with respect to the other categories (Table 2). All tetracycline resistant strains carried both the *tet*(M) and *tet*(L) genes, and all but one (i.e., *E*. *faecium* 39 BA) streptomycin resistant strain carried the gene *ant*(6’)-Ia (Table 2). The co-presence on mobile plasmids of *erm*(B), *tet*(M), *tet*(L) and/or *ant*(6’)-Ia (i.e *aadE*) has been reported both in human and food enterococcal isolates [55, 58, 59]. The finding of gentamicin resistance only among omnivore strains could be related to the use of aminoglycosides in veterinary medicine.

**Table 2:** Resistance phenotype and genotype of the erythromycin resistant isolates carrying *erm*(B).

Strain		Resistance phenotype	Resistance genotype
**omnivore**			
36PA	*E*. *faecalis*	ERY, CN, TET	*erm*(B), *aac-aph*, *tet*(M), *tet*(L)
36BA	*E*. *faecalis*	ERY, STR, DAP, TET	*erm*(B), *ant*(6’)-Ia, *tet*(M), *tet*(L)
37TO	*E*. *faecalis*	ERY, CN, TET	*erm*(B), *aac-aph*, *tet*(M), *tet*(L)
28BA	*E*. *faecium*	ERY, STR, DAP, TET	*erm*(B), *ant*(6’)-Ia, *tet*(M), *tet*(L)
37BA	*E*. *faecium*	ERY, STR, DAP, TET	*erm*(B), *ant*(6’)-Ia, *tet*(M), *tet*(L)
39BA	*E*. *faecium*	ERY, STR, TET	*erm*(B), *tet*(M), *tet*(L)
32BA	*E*. *faecium*	ERY, TET	*erm*(B), *tet*(M), *tet*(L)
33TO	*E*. *avium*	ERY, STR, CN, TET	*erm*(B), *erm*(A), *ant*(6’)-Ia, *aac-aph*, *tet*(M), *tet*(L)
**ovo-lacto vegetarian**			
13BA	*E*. *faecalis*	ERY, STR, TET	*erm*(B), *ant*(6’)-Ia, *tet*(M), *tet*(L)
17aBO	*E*. *faecium*	ERY, Q/D, TET	*erm*(B), *tet*(M), *tet*(L)
01BA	*E*. *hirae*	ERY, AMP, STR,	*erm*(B), *ant*(6’)-Ia
05bPA	*E*. *durans*	ERY, STR, TET	*erm*(B), *ant*(6’)-Ia, *tet*(M), *tet*(L)
**vegan**			
16BA	*E*. *faecalis*	ERY, DAP	*erm*(B)
24TO	*E*. *faecalis*	ERY, TET	*erm*(B), *tet*(M), *tet*(L)
27TO	*E*. *faecalis*	ERY, TET	*erm*(B), *tet*(M), *tet*(L)
24BA	*E*. *avium*	ERY, STR, TET	*erm*(B), *ant*(6’)-Ia, *tet*(M), *tet*(L)
35PA	*E*. *avium*	ERY, STR	*erm*(B), *ant*(6’)-Ia
21TO	*S*. *pasteurianus*	ERY, TET	*erm*(B), *tet*(M), *tet*(L)
25TO	*S*. *salivarius*	ERY, TET	*erm*(B), *tet*(M), *tet*(L)

ERY, erythromycin; AMP, ampicillin; CN, gentamicin; STR, streptomycin; LEV, levofloxacin; Q/D, quinupristin/dalfopristin; LZD, linezolid; VAN, vancomycin; DAP, daptomycin; TET, tetracycline

### Conjugation experiments

Two resistant strains belonging to different species and selected from each category (omnivore, ovo-lacto vegetarian or vegan) of subjects were used as donors in mating experiments with the two recipients *E*. *faecalis* JH2-2 and *E*. *faecium* 64/3. Transconjugants were obtained from all the donors except for *E*. *faecalis* 37TO (omnivore) and *E*. *hirae* 01BA (ovo-lacto vegetarian). Transfer frequencies and resistance genes acquired from the donors *E*. *faecium* 37BA, *E*. *faecalis* 13BA, *E*. *avium* 35PA and the *E*. *faecalis* 27TO are reported in Table 3. All these donors transferred *erm*(B) to the recipient *E*. *faecalis* JH2-2 with different frequencies (5.7 x 10^−9^ to 1.7 x 10^−6^) and not to *E*. *faecium* 64/3, except for the donor *E*. *faecium* 37BA. Transfer frequencies were higher in the intraspecific mating assays confirming previous results [39] and the highest frequency was observed with the donor *E*. *faecium* 37BA and the recipient *E*. *faecium* 64/3 (5.7 x 10^−4^). All transconjugants maintained erythromycin resistance and *erm*(B) gene even after repeated passages in antibiotic-free medium. In addition, in a previous study [55], *E*. *faecium* 37BA was able to transfer both *erm*(B) and *tet*(M)-*tet*(L) genes to the recipients *E*. *faecium* 64/3 and *Listeria welshimeri* 11857RF, with frequencies of 5.7 x 10^−4^ and 8.5 x 10^−8^, respectively. In these transconjugants, also stability of the whole plasmid (pEf37BA) carrying *erm*(B) and additional AR genes, was demonstrated [55].

**Table 3:** Mating experiments and transfer frequencies.

	Donor strain	Recipient	Transconjugants Transfer frequency	Resistance genes transferred
**omnivore**	*E*. *faecalis* 37TO	*E*. *faecalis* JH2-2	< 1 x 10^−9^	-
		*E*. *faecium* 64/3	< 1 x 10^−9^	-
	*E*. *faecium* 37BA	*E*. *faecalis* JH2-2	1.7 ± 0.43 x 10^−6^	*erm*(B)
		*E*. *faecium* 64/3*	5.7 ± 0.35 x 10^−4^	*erm*(B), *tet*(M), *tet*(L)
**ovo-lacto vegetarian**	*E*. *faecalis* 13BA	*E*. *faecalis* JH2-2	7.9 ± 0.27 x 10^−6^	*erm*(B)
		*E*. *faecium* 64/3	< 1 x 10^−9^	-
	*E*. *hirae* 01BA	*E*. *faecalis* JH2-2	< 1 x 10^−9^	-
		*E*. *faecium* 64/3	< 1 x 10^−9^	-
**vegan**	*E*. *avium* 35PA	*E*. *faecalis* JH2-2	5.7 ± 0.41 x 10^−9^	*erm*(B)
		*E*. *faecium* 64/3	< 1 x 10^−9^	
	*E*. *faecalis* 27TO	*E*. *faecalis* JH2-2	2 ± 0.6 x 10^−8^	*erm*(B)
		*E*. *faecium* 64/3	< 1 x 10^−9^	

*The mating *E*. *faecium* 37BA x *E*. *faecium* 64/3 was performed in a previous study [57]

The recovery of commensal MDR strains from the feces of healthy individuals and the ability of these strains to transfer the relevant resistance genes *in vitro* suggests the potential role of the human gut microbiota as a reservoir of transferable AR genes. Indeed, the horizontal gene transfer of AR genes from one bacterium to another in the gut environment has been documented [60]. Our findings are in line with the reported increase in the incidence of antibiotic resistant strains carried by healthy humans [15, 61] and their long-term persistence in the gut [12]. Resistant commensal enterococci harboring the *erm*(B) gene could derive from contaminated foods of animal or vegetable origins [62] or could have been selected during antibiotic therapy and persisted for years after treatment in the absence of antibiotic pressure [63].

## Conclusions

Dietary food intake represents the main reservoir of antibiotic resistant bacteria and their genes in the human gut. The effect of long term vegan, ovo-lacto vegetarian or omnivore diets on the prevalence of erythromycin-resistant LAB and their genes in the human gut was studied. Eleven different species of erythromycin-resistant LAB from *Enterococcus* and *Streptococcus* genera were recovered from the fecal samples obtained from each dietary group, showing that diet did not significantly affect the occurrence of erythromycin-resistant bacteria in the human gut. However, LAB isolates from omnivores were resistant to a greater number of tested antibiotics compared to isolates from ovo-lacto vegetarians and vegans. This could be related to the extensive use of antibiotics in farm animals for therapeutic or prophylactic purposes. Finally, the ability of commensal MDR strains to transfer resistance genes *in vitro* is of great concern since they may act as reservoirs of AR genes and be the source of their spreading to other strains, including human pathogens.

## Ethics and informed consent

All participants were informed about the aims of the study and provided informed written consent. The study was approved by the Ethics Committee of (i) Azienda Sanitaria Locale (Bari) (protocol N.1050), (ii) Azienda Ospedaliera Universitaria of Bologna (protocol N.0018396), (iii) Province of Parma (protocol N.22884) and (iv) University of Torino (protocol N.1/2013/C).

## Supporting information

S1 Table Complete list and characteristics of the isolates obtained from the feces of the volunteers following vegan, ovo-lacto vegetarian and omnivore diet. ^a^ Percentage of identical nucleotides in the sequence obtained from the isolate and the sequence of the closest relative found in the GenBank database; ^b^ Accession number of the sequence of the closest relative found by a BLAST search; * One or more isolates from each ARDRA group were sequenced and identified; ^T^ Type strain; MIC, Minimum Inhibitory Concentration.
(DOCX)

S2 Table Results of statistical analyses. (PDF)
