# Supplementary material for: Erythromycin-resistant lactic acid bacteria in the healthy gut of vegans, ovo-lacto vegetarians and omnivores
Source: PLoS One. 2019 Aug 2;14(8):e0220549. doi: 10.1371/journal.pone.0220549 (PMC6677300; doi:10.1371/journal.pone.0220549)
Supplement: S1 Table — a Percentage of identical nucleotides in the sequence obtained from the isolate and the sequence of the closest relative found in the GenBank database; b Accession number of the sequence of the closest relative found by a BLAST search; * One or more isolates from each ARDRA group were sequenced and identified; T Type strain; MIC, Minimum Inhibitory Concentration. (DOCX) [file pone.0220549.s001.docx]

**S1 Table:** **Complete list and characteristics of the isolates obtained from the feces of the volunteers following vegan, ovo-lacto vegetarian and omnivore diet.**

|  |  |  |  |  |  | **Erythromycin resistance genes** | | |
| --- | --- | --- | --- | --- | --- | --- | --- | --- |
| **Sample** | **Diet** | **Species** | **% Identity^a^ *** | **Accession number^b^ *** | **MIC (mg L^-1^)** | ***erm*(A)** | ***erm*(B)** | ***erm*(C)** |
| 15BA | Vegan | *Enterococcus pallens* | 99 | NR_113933 ^T^ | 16 | - | - | - |
| 16BA | Vegan | *Enterococcus faecalis* | 99 | AB681178 ^T^ | > 256 | - | + | - |
| 17BA | Vegan | *Enterococcus faecalis* |  |  | 2 | - | - | - |
| 19BA | Vegan | *Enterococcus faecium* |  |  | 4 | - | - | - |
| 20BA | Vegan | *Enterococcus faecium* | 99 | AJ420800 ^T^ | ≤ 0.25 | - | - | - |
| 21BA | Vegan | *Enterococcus faecium* |  |  | 32 | - | - | - |
| 23BA | Vegan | *Enterococcus durans* |  |  | ≤ 0.25 | - | - | - |
| 24BA | Vegan | *Enterococcus avium* | 99 | AB681175 ^T^ | 128 | - | + | - |
| 25BA | Vegan | *Enterococcus faecium* |  |  | 16 | - | - | - |
| 26BA | Vegan | *Enterococcus faecium* |  |  | 16 | - | - | - |
| 02BO | Vegan | *Enterococcus faecium* |  |  | 16 | - | - | - |
| 07BO | Vegan | *Enterococcus durans* |  |  | 8 | - | - | - |
| 10BO | Vegan | *Enterococcus faecium* |  |  | 32 | - | - | - |
| 12aBO | Vegan | *Enterococcus faecalis* |  |  | 2 | - | - | - |
| 12bBO | Vegan | *Enterococcus faecalis* | 99 | AB681178 ^T^ | 2 | - | - | - |
| 12cBO | Vegan | *Enterococcus faecium* |  |  | 4 | - | - | - |
| 18BO | Vegan | *Enterococcus faecium* |  |  | 32 | - | - | - |
| 26BO | Vegan | *Enterococcus durans* | 99 | AB596943 ^T^ | 16 | - | - | - |
| 35bBO | Vegan | *Enterococcus faecium* |  |  | 32 | - | - | - |
| 36aBO | Vegan | *Enterococcus durans* |  |  | 32 | - | - | - |
| 36bBO | Vegan | *Enterococcus faecalis* | 99 | AB681178 ^T^ | 0.5 | - | - | - |
| 44aBO | Vegan | *Enterococcus faecium* |  |  | ≤ 0.25 | - | - | - |
| 44bBO | Vegan | *Enterococcus faecium* | 99 | LC071831 ^T^ | 32 | - | - | - |
| 07bPA | Vegan | *Enterococcus durans* | 99 | AB596943 ^T^ | 16 | - | - | - |
| 09PA | Vegan | *Enterococcus durans* |  |  | 4 | - | - | - |
| 11PA | Vegan | *Enterococcus durans* | 99 | AB596943 ^T^ | 16 | - | - | - |
| 12aPA | Vegan | *Enterococcus durans* |  |  | 0.5 | - | - | - |
| 12bPA | Vegan | *Enterococcus durans* |  |  | ≤ 0.25 | - | - | - |
| 17aPA | Vegan | *Enterococcus durans* |  |  | 16 | - | - | - |
| 17bPA | Vegan | *Enterococcus faecium* |  |  | 16 | - | - | - |
| 21aPA | Vegan | *Enterococcus faecium* | 99 | AB681184 ^T^ | 8 | - | - | - |
| 21bPA | Vegan | *Enterococcus faecium* |  |  | ≤ 0.25 | - | - | - |
| 22aPA | Vegan | *Enterococcus faecium* |  |  | 16 | - | - | - |
| 22bPA | Vegan | *Enterococcus faecium* |  |  | 16 | - | - | - |
| 26bPA | Vegan | *Enterococcus faecium* |  |  | ≤ 0.25 | - | - | - |
| 26cPA | Vegan | *Enterococcus faecium* |  |  | 2 | - | - | - |
| 32aPA | Vegan | *Enterococcus faecium* | 99 | AB681184 ^T^ | 16 | - | - | - |
| 32bPA | Vegan | *Enterococcus faecium* |  |  | 16 | - | - | - |
| 33PA | Vegan | *Enterococcus faecium* |  |  | 16 | - | - | - |
| 35PA | Vegan | *Enterococcus avium* | 99 | AB681175 ^T^ | > 256 | - | + | - |
| 03fTO | Vegan | *Streptococcus parasanguinis* | 99 | AY485605 ^T^ | 8 | - | - | - |
| 07aTO | Vegan | *Enterococcus faecium* |  |  | 8 | - | - | - |
| 07bTO | Vegan | *Enterococcus casseliflavus* | 99 | AB681176 ^T^ | ≤ 0.25 | - | - | - |
| 07cTO | Vegan | *Enterococcus casseliflavus* |  |  | 16 | - | - | - |
| 10TO | Vegan | *Enterococcus faecium* |  |  | 16 | - | - | - |
| 11TO | Vegan | *Enterococcus faecium* |  |  | 16 | - | - | - |
| 19TO | Vegan | *Enterococcus faecium* |  |  | 16 | - | - | - |
| 21TO | Vegan | *Streptococcus pasteurianus* | 99 | DQ232528 ^T^ | > 256 | - | + | - |
| 24TO | Vegan | *Enterococcus faecalis* | 99 | LC071830 ^T^ | > 256 | - | + | - |
| 25TO | Vegan | *Streptococcus salivarius* | 99 | LN623639 | > 256 | - | + | - |
| 27TO | Vegan | *Enterococcus faecalis* | 99 | LC096215 ^T^ | > 256 | - | + | - |
| 32TO | Vegan | *Enterococcus pallens* | 99 | AB681228 ^T^ | 64 | - | - | - |
| 35TO | Vegan | *Enterococcus durans* |  | AB596943 ^T^ | 16 | - | - | - |
| 44TO | Vegan | *Enterococcus faecium* |  |  | 32 | - | - | - |
| 01BA | Vegetarian | *Enterococcus hirae* | 99 | AB680022^T^ | 32 | - | + | - |
| 02BA | Vegetarian | *Enterococcus faecium* | 99 | AB681184 ^T^ | 16 | - | - | - |
| 03BA | Vegetarian | *Enterococcus durans* | 99 | AB596943 ^T^ | 32 | - | - | - |
| 04BA | Vegetarian | *Enterococcus faecium* |  |  | 16 | - | - | - |
| 05BA | Vegetarian | *Enterococcus faecium* | 99 | AJ420800 ^T^ | 2 | - | - | - |
| 07BA | Vegetarian | *Enterococcus faecium* |  |  | 2 | - | - | - |
| 08BA | Vegetarian | *Enterococcus faecium* |  |  | 2 | - | - | - |
| 09BA | Vegetarian | *Enterococcus faecium* |  |  | 2 | - | - | - |
| 10BA | Vegetarian | *Enterococcus faecium* |  |  | 4 | - | - | - |
| 11BA | Vegetarian | *Enterococcus faecium* | 99 | AB681184 ^T^ | 0.5 | - | - | - |
| 12BA | Vegetarian | *Enterococcus faecium* |  |  | 2 | - | - | - |
| 13BA | Vegetarian | *Enterococcus faecalis* | 99 | AB681178 ^T^ | > 256 | - | + | - |
| 01BO | Vegetarian | *Enterococcus durans* |  |  | 16 | - | - | - |
| 04BO | Vegetarian | *Enterococcus faecium* | 99 | AB681184 ^T^ | 2 | - | - | - |
| 05aBO | Vegetarian | *Enterococcus faecium* |  |  | 1 | - | - | - |
| 05bBO | Vegetarian | *Enterococcus faecium* |  |  | 32 | - | - | - |
| 17aBO | Vegetarian | *Enterococcus faecium* | 99 | AB681184 ^T^ | > 256 | - | + | - |
| 19BO | Vegetarian | *Enterococcus faecium* |  |  | 4 |  |  |  |
| 25BO | Vegetarian | *Enterococcus durans* |  |  | 2 | - | - | - |
| 27BO | Vegetarian | *Enterococcus faecium* |  |  | 4 | - | - | - |
| 32BO | Vegetarian | *Enterococcus durans* | 99 | AB596943 ^T^ | 8 | - | - | - |
| 34BO | Vegetarian | *Enterococcus faecium* |  |  | 8 | - | - | - |
| 37BO | Vegetarian | *Enterococcus faecium* |  |  | 4 | - | - | - |
| 39BO | Vegetarian | *Enterococcus faecium* |  |  | 32 | - | - | - |
| 40BO | Vegetarian | *Enterococcus durans* |  |  | 16 | - | - | - |
| 02PA | Vegetarian | *Enterococcus durans* |  |  | 4 | - | - | - |
| 03PA | Vegetarian | *Enterococcus durans* | 99 | AB596943 ^T^ | 8 | - | - | - |
| 04PA | Vegetarian | *Enterococcus faecium* | 99 | AB681184 ^T^ | 16 | - | - | - |
| 05bPA | Vegetarian | *Enterococcus durans* | 99 | AB596943 ^T^ | > 256 | - | + | - |
| 06PA | Vegetarian | *Enterococcus faecium* |  |  | 1 | - | - | - |
| 08PA | Vegetarian | *Enterococcus durans* | 99 | AB596943 ^T^ | 16 | - | - | - |
| 10PA | Vegetarian | *Enterococcus durans* |  |  | 4 | - | - | - |
| 13aPA | Vegetarian | *Enterococcus durans* |  |  | 16 | - | - | - |
| 13bPA | Vegetarian | *Enterococcus faecium* |  |  | 0.5 | - | - | - |
| 14aPA | Vegetarian | *Enterococcus faecium* | 99 | AB681184 ^T^ | 16 | - | - | - |
| 14bPA | Vegetarian | *Enterococcus faecium* |  |  | 16 | - | - | - |
| 27aPA | Vegetarian | *Enterococcus faecium* |  |  | 32 | - | - | - |
| 27bPA | Vegetarian | *Enterococcus faecium* |  |  | ≤ 0.25 | - | - | - |
| 30PA | Vegetarian | *Enterococcus faecium* |  |  | 16 | - | - | - |
| 01TO | Vegetarian | *Enterococcus faecium* | 99 | KP100399 | 32 | - | - | - |
| 01fTO | Vegetarian | *Enterococcus faecium* |  |  | 32 | - | - | - |
| 02TO | Vegetarian | *Enterococcus faecium* |  |  | 32 | - | - | - |
| 04TO | Vegetarian | *Enterococcus faecium* |  |  | 16 | - | - | - |
| 06TO | Vegetarian | *Enterococcus faecium* |  |  | 16 | - | - | - |
| 26TO | Vegetarian | *Enterococcus faecium* |  |  | 0.5 | - | - | - |
| 28TO | Vegetarian | *Enterococcus faecium* |  |  | 0.5 | - | - | - |
| 29TO | Vegetarian | *Enterococcus faecium* |  |  | 0.5 | - | - | - |
| 38TO | Vegetarian | *Enterococcus durans* |  |  | 64 | - | - | - |
| 39TO | Vegetarian | *Enterococcus faecium* |  |  | 64 | - | - | - |
| 41TO | Vegetarian | *Enterococcus mundtii* | 99 | AB681188 ^T^ | 0.5 | - | - | - |
| 42TO | Vegetarian | *Enterococcus faecium* |  |  | 4 | - | - | - |
| 28BA | Omnivore | *Enterococcus faecium* | 99 | AJ420800 ^T^ | >256 | - | + | - |
| 29BA | Omnivore | *Enterococcus faecium* |  |  | 8 | - | - | - |
| 30BA | Omnivore | *Enterococcus faecium* |  |  | 2 | - | - | - |
| 31BA | Omnivore | *Enterococcus faecium* |  |  | 2 | - | - | - |
| 32BA | Omnivore | *Enterococcus faecium* | 99 | AB681184 ^T^ | >256 | - | + | - |
| 33BA | Omnivore | *Enterococcus faecium* |  |  | 16 | - | - | - |
| 34BA | Omnivore | *Enterococcus durans* | 99 | GQ337025 ^T^ | 1 | - | - | - |
| 35BA | Omnivore | *Enterococcus faecium* | 99 | AJ420800 ^T^ | 0.5 | - | - | - |
| 36BA | Omnivore | *Enterococcus faecalis* | 99 | AB681178 ^T^ | >256 | - | + | - |
| 37BA | Omnivore | *Enterococcus faecium* | 99 | AJ420800 ^T^ | >256 | - | + | - |
| 38aBA | Omnivore | *Enterococcus faecium* |  |  | 64 | - | - | - |
| 38bBA | Omnivore | *Enterococcus durans* |  |  | 64 | - | - | - |
| 39BA | Omnivore | *Enterococcus faecium* |  |  | >256 | - | + | - |
| 40BA | Omnivore | *Enterococcus durans* | 99 | AB596943 ^T^ | 1 | - | - | - |
| 41BA | Omnivore | *Enterococcus faecium* |  |  | 4 | - | - | - |
| 03BO | Omnivore | *Enterococcus faecium* |  |  | 32 | - | - | - |
| 06aBO | Omnivore | *Enterococcus faecium* |  |  | 1 | - | - | - |
| 06bBO | Omnivore | *Enterococcus faecium* |  |  | 2 | - | - | - |
| 08BO | Omnivore | *Enterococcus faecium* |  |  | 16 | - | - | - |
| 09BO | Omnivore | *Enterococcus faecium* |  |  | 4 | - | - | - |
| 16BO | Omnivore | *Enterococcus faecium* | 99 | LC071831 ^T^ | 16 |  |  |  |
| 20BO | Omnivore | *Enterococcus faecium* | 99 | AJ420800 ^T^ | 2 | - | - | - |
| 22BO | Omnivore | *Enterococcus durans* | 98 | KF029506 ^T^ | 8 | - | - | - |
| 23BO | Omnivore | *Enterococcus durans* |  |  | 32 | - | - | - |
| 24BO | Omnivore | *Enterococcus durans* | 99 | AB596943 ^T^ | 8 | - | - | - |
| 28BO | Omnivore | *Enterococcus durans* |  |  | 4 | - | - | - |
| 29BO | Omnivore | *Enterococcus durans* | 99 | AB596943 ^T^ | 8 | - | - | - |
| 30BO | Omnivore | *Enterococcus faecium* |  |  | 8 | - | - | - |
| 42BO | Omnivore | *Enterococcus faecium* |  |  | 32 | - | - | - |
| 01PA | Omnivore | *Enterococcus durans* |  |  | 8 | - | - | - |
| 25PA | Omnivore | *Enterococcus faecalis* | 99 | AB681178 ^T^ | 0.5 | - | - | - |
| 29PA | Omnivore | *Enterococcus faecium* |  |  | 4 | - | - | - |
| 31PA | Omnivore | *Enterococcus faecium* |  |  | 4 | - | - | - |
| 36PA | Omnivore | *Enterococcus faecalis* |  |  | >256 | - | + | - |
| 37PA | Omnivore | *Enterococcus faecium* |  |  | 8 | - | - | - |
| 38PA | Omnivore | *Enterococcus faecium* |  |  | 8 | - | - | - |
| 39PA | Omnivore | *Enterococcus faecium* |  |  | 8 | - | - | - |
| 13TO | Omnivore | *Enterococcus faecium* |  |  | 2 | - | - | - |
| 13fTO | Omnivore | *Enterococcus faecium* |  |  | 8 | - | - | - |
| 14TO | Omnivore | *Enterococcus faecium* | 99 | AJ420800 ^T^ | 4 | - | - | - |
| 15TO | Omnivore | *Enterococcus faecium* |  |  | 4 | - | - | - |
| 16TO | Omnivore | *Enterococcus faecium* |  |  | 2 | - | - | - |
| 17TO | Omnivore | *Enterococcus faecium* |  |  | 16 | - | - | - |
| 18TO | Omnivore | *Enterococcus faecium* |  |  | 8 | - | - | - |
| 22TO | Omnivore | *Enterococcus faecium* |  |  | 16 | - | - | - |
| 23TO | Omnivore | *Enterococcus faecium* |  |  | 1 | - | - | - |
| 31TO | Omnivore | *Streptococcus parasanguinis* | 99 | AY485605 ^T^ | 4 | - | - | - |
| 33TO | Omnivore | *Enterococcus avium* | 99 | AB681175 ^T^ | >256 | + | + | - |
| 37TO | Omnivore | *Enterococcus faecalis* | 99 | LC096215 ^T^ | >256 | - | + | - |
| 43TO | Omnivore | *Enterococcus faecium* |  |  | 8 | - | - | - |

^a^ Percentage of identical nucleotides in the sequence obtained from the isolate and the sequence of the closest relative found in the GenBank database; ^b^ Accession number of the sequence of the closest relative found by a BLAST search; * One or more isolates from each ARDRA group were sequenced and identified; ^T^ Type strain; MIC, Minimum Inhibitory Concentration
