# Supplementary material for: Erythromycin-resistant lactic acid bacteria in the healthy gut of vegans, ovo-lacto vegetarians and omnivores
Source: PLoS One. 2019 Aug 2;14(8):e0220549. doi: 10.1371/journal.pone.0220549 (PMC6677300; doi:10.1371/journal.pone.0220549)
Supplement: S2 Table — (PDF) [file pone.0220549.s002.pdf]

S2 Table: Results of statistical analyses

observed frequencies

	Vegans	Vegetarians	Omnivores	TOT	P value
Enterococcus faecium	27	36	35	98	0,3173783
Enterococcus faecalis	7	1	4	12	0,1286923
Enterococcus durans	11	12	9	32	0,8274515
Enterococcus avium	2	0	1	3	n.a.
Enterococcus hirae	0	1	0	1	n.a.
Enterococcus pallens	2	0	0	2	n.a.
Enterococcus mundtii	0	1	0	1	n.a.
Enterococcus casseliflavus	2	0	0	2	n.a.
Streptococcus parasanguinis	1	0	1	2	n.a.
Streptococcus pasteurianus	1	0	0	1	n.a.
Streptococcus salivarius	1	0	0	1	n.a.
TOT	54	51	50	155	

n.a. = not applicable

expected frequencies

	Vegans	Vegetarians	Omnivores
Enterococcus faecium	34,14	32,24	31,61
Enterococcus faecalis	4,18	3,94	3,87
Enterococcus durans	11,14	10,52	10,32
Enterococcus avium	1,04	0,98	0,96
Enterococcus hirae	0,34	0,32	0,32
Enterococcus pallens	0,69	0,65	0,64
Enterococcus mundtii	0,34	0,32	0,32
Enterococcus casseliflavus	0,69	0,65	0,64
Streptococcus parasanguinis	0,69	0,65	0,64
Streptococcus pasteurianus	0,34	0,32	0,32
Streptococcus salivarius	0,34	0,32	0,32
Pvalue	0,2445328	0,503839384	0,973429442

observed frequencies

**ery**	Vegans	vegetarians	omnivores	TOT	Pvalue
res	38	28	31	97	0,6046155
sens	16	23	19	58	
TOT	54	51	50	155	

expected frequencies

**ery**	Vegans	vegetarians	omnivores
res	33,79	31,91	31,29
sens	20,2	19,08	18,7
Pvalue	0,2370916	0,257070034	0,93098472

observed frequencies

**ermB**	Vegans	vegetarians	omnivores	TOT	Pvalue
pos	7	4	8	19	0,5898862
neg	31	24	23	78	
TOT	38	28	31	97	

expected frequencies

**ermB**	Vegans	vegetarians	omnivores
pos	7,44	5,4	6
neg	30,5	22,5	24,9
Pvalue	0,8532434	0,496242474	0,36763388
